# Impacts of garlic extract on testicular oxidative stress and sperm characteristics in type 1 and 2 diabetic rats: An experimental study

**DOI:** 10.18502/ijrm.v19i10.9825

**Published:** 2021-11-04

**Authors:** Fatemeh Lotfi, Nasrin Ziamajidi, Roghayeh Abbasalipourkabir, Mohammad Taghi Goodarzi, Sara Soleimani Asl

**Affiliations:** ^1^Department of Clinical Biochemistry, School of Medicine, Hamadan University of Medical Sciences, Hamadan, Iran.; ^2^Molecular Medicine Research Center, Hamadan University of Medical Sciences, Hamadan, Iran.; ^3^Department of Biochemistry, Shahrood Branch, Islamic Azad University, Shahrood, Iran.; ^4^Department of Anatomy, School of Medicine, Hamadan University of Medical Sciences, Hamadan, Iran.

**Keywords:** Diabetes mellitus, Garlic, Oxidative stress, Inflammation, Testis.

## Abstract

**Background:**

Hyperglycemia damages various tissues such as the testes through oxidative stress and inflammation, which can eventually lead to infertility.

**Objective:**

Garlic extract effects on the testicular tissue of diabetic rats were investigated.

**Materials and Methods:**

In this experimental study, 36 male Wistar rats (8-wk old, weighing 230-300 gr) were randomly divided into 6 groups (n = 6/each) including; C: control rats, G: received 0.4 gr of garlic extract/100 gr body weight, D1: Streptozotocin-induced-diabetic rats or type 1, D1+G: D1 rats that were treated with garlic, D2: Streptozotocin + nicotinamide-induced-diabetic rats or type 2, D2+G: D2 rats treated with garlic. At the end of the study, serum testosterone was assayed by ELISA. Also, sperm quality and quantity were evaluated. For determination of oxidative stress status, total antioxidant capacity, total oxidative status, lipid peroxidation, and thiol groups were assayed in the testis tissues of the rats by colorimetric methods. Also, inducible nitric oxide synthase (*iNOS*) gene expression and the protein level of interleukin-1 β (IL-1 β ) were determined by quantitative real-time polymerase chain reaction and enzyme-linked immunosorbent assay, respectively.

**Results:**

In diabetic rats, glucose, total oxidative status and lipid peroxidation, *iNOS* gene expression, and IL-1 β were higher than in non-diabetic rats, whereas testosterone, total antioxidant capacity and thiol groups, and sperm quality were significantly lower compared with control rats. These alterations were normalized by garlic intervention.

**Conclusion:**

In diabetic rats, garlic was associated with reduced glucose, oxidative stress, IL-1 β , and *iNOS* gene expression and increased testosterone and sperm quality. So, the results suggest that garlic can reduce the severity of damage in testicular tissues of diabetic rats through its hypoglycemic, antioxidant, and anti-inflammatory properties

## 1. Introduction

Diabetes mellitus (DM) is one of the most prevalent metabolic disorders threatening human health worldwide. It is associated with the impaired functioning and structure of various organs, such as the heart, liver, kidney, eye, and testis (1). In diabetes, testicular dysfunction, abnormal histology of testes, and infertility have been observed (2). Persistent hyperglycemia in DM increases the production of reactive oxygen species (ROS) and nitric oxide (NO) and induces oxidative stress. ROS and NO cause cell apoptosis in the testes, atrophy of sex organs, decreased sperm quality, and decreased levels of serum sex hormones such as testosterone, luteinizing hormone, and follicle-stimulating hormone in male animal models.

The decline in the concentration of testosterone in diabetic patients and experimental animals can lead to a significant decrease in accessory sex gland weight, reduced epididymis sperm content, erectile dysfunction, and a reduction in sperm motility and semen volume (3). Increased levels of free radicals in DM cause nuclear factor (NF)-κB to be activated, followed by increased expression of other genes such as inducible nitric oxide synthase (*iNOS*) and inflammatory cytokines such as interleukin-1 β (IL-1 β ). This process may cause tissue damage, inflammation, and apoptosis (4).

Since diabetic complications arise from chronic hyperglycemia, any substance that reduces glucose levels can lead to the improvement of tissue damage. In the past, the use of medicinal plants has been considered in the treatment of DM (5). *Allium sativum *(garlic) is one of the most common pharmaceutical plants and its hypoglycemic and antioxidant properties have been demonstrated in several studies (6). In this study, for the first time we investigated the effects of aqueous garlic extract on oxidative stress, inflammation and testicular function in two models of diabetic rats (Streptozotocin-induced or type 1 and Streptozotocin+nicotinamide-induced or type 2).

## 2. Materials and Methods

### Preparation of aqueous garlic extract

Garlic was purchased from a local market in Hamadan, Iran. Garlic bulbs were thoroughly peeled, washed, and cut into small pieces. To prepare the aqueous garlic extract, 100 gr of garlic was homogenized in 250 ml of distilled water. After filtration with Whatman no. 1 filter paper (Camlab, UK), 1 mL of this fresh solution/100 gr body weight, equivalent to 0.4 gr/100 gr body weight, was given by gavage for the treatment of the rats (6).

### Animals and experimental design

This experimental study was conducted at Hamadan University of Medical Sciences, Hamadan, Iran, and performed on 36 adult male Wistar rats (8-wk old, weighing 230-300 gr) which were acquired from Hamadan University of Medical Sciences, Hamadan, Iran between 2018 and 2020.

The rats were randomly divided into six groups (n = 6/group). Streptozotocin (STZ) (Sigma, USA) alone and STZ + nicotinamide (Sigma, USA) were used for the induction of diabetes types 1 and 2, respectively.

Group C: Control rats that received a single dose of citrate buffer (0.1 M, pH 4.5).

Group G: Rats that received only aqueous garlic extract (0.4 g/100 g body weight/day, by gavage, for 5 wk).

Group D1: To design a diabetes type 1 model, rats were injected with 65 mg/kg STZ dissolved in 0.1 M citrate buffer with pH = 4.5. The glucose level of rats' blood was determined by a strip-operated blood glucose sensor (Accuchek; Roche, Germany) on the fifth day of injection of STZ. The glucose levels above 300 mg/dl indicated diabetes type 1.

D1 + G: STZ-induced diabetic rats that were treated with aqueous garlic extract (0.4 g/100 g body weight/day, by gavage, for 5 wk).

Group D2: To design a diabetes type 2 model, rats were injected with 65 mg/kg STZ dissolved in 0.1 M citrate buffer with pH = 4.5 followed by 110 mg/kg nicotinamide 15 min later. The glucose level of rats' blood was determined by a strip-operated blood glucose sensor (Accuchek; Roche, Germany) on the fifth day of injection of STZ. The glucose levels above 250 mg/dl indicated diabetes type 2.

Group D2 + G: STZ + nicotinamide-induced diabetic rats were treated with aqueous garlic extract (0.4 g/100g body weight/day, by gavage, for 5 wk). At the end of the 5-wk treatment (7-9), animals were weighed and killed. The serum samples were collected and stored at -20°C for testosterone assay. One testis was removed quickly, washed by phosphate-buffered saline, snap-frozen in liquid nitrogen, and stored at -80°C for molecular and biochemical analysis. The second testis was fixed in 10% formalin for histopathological analysis.

### Serum glucose and testosterone assay

The glucose level in the serum was assayed by colorimetric methods (Pars Azmoon, Tehran, Iran). The serum concentration of total testosterone was determined using the enzyme-linked immunosorbent assay (ELISA) method (Cayman, USA).

### Determination of oxidative stress status in testis tissues of rats

For the preparation of testis tissue homogenates, liquid nitrogen was used. The homogenates were lysed by lysis buffer including 10 mM KCl, 1.5 mM MgCl ${}_{2}$ , 1 mM EDTA, 0.1% Triton X-100, 10 mM HEPES, 0.5 mM DTT, protease inhibitor cocktail, pH 7.9. All procedures were done on ice. Then the homogenates were centrifuged by refrigerated centrifuge at 10,000 g for 15 min at 4°C. The supernatants were separated and stored for future experiments. The total protein levels of testis homogenate samples were determined by the Bradford method (10).

The ferric-reducing antioxidant potential method based on the Benzie and Strain procedure was used to measure the total antioxidant capacity (TAC) in the tissue homogenates of rats' testis. Ferric tripyridyl triazine was reduced to the blue-colored ferrous form at low pH and the absorption was measured at 593 nm. A solution of Fe (II) concentration (FeSO4.7H2O) was used as a standard (11).

Evaluation of the total oxidative status (TOS) was based on the oxidation of Fe (II) to Fe (III) in the presence of oxidant factors at low pH (FOX1 assay). Discernment was according to the colored complex formation between the produced Fe (III) and xylenol orange and the absorption was measured at 560 nm. Hydrogen peroxide was used for the calibration of this assay (12).

The evaluation of lipid peroxidation (LPO) was based on the formation of aldehydes during LPO in testis tissue homogenate through reaction with TBA. The fluorescence intensity of its product was measured at 515 nm excitation and 553 nm emission wavelength. As a standard solution, tetraethoxy propane was used (13).

The total thiol concentration assay was measured in accordance with Ellman. This assay is based on 2, 2-dithiobisnitrobenzoic acid, which is reduced by SH groups to form a 2-nitro-5-mercaptobenzoic acid anion. This complex yellow color had an absorption maxima at 412 nm (14).

### Real-time qPCR analyses for *iNOS*

RNX-Plus reagent (CinnaGen, Iran) was used for total RNA extraction from testis tissues. For determination of the quantity and purity of the RNA samples, a Nano Drop spectrophotometer (Thermo Fisher Scientific, USA) was used, and for evaluation of the RNA integrity, agarose gel electrophoresis was used.

The total extracted RNA was transcribed reversely to cDNA by a Revert Aid ${}^{\mathrm{TM}}$ First Strand cDNA synthesis kit (Thermo Scientific, Lithuania). The SYBR Premix (AMPLICON, Denmark) on a Light CyclerⓇ 96 System (Roche Life Science, Deutschland GmbH Sandhofer, Germany) was applied for quantitative real-time polymerase chain reaction (qRT-PCR).

The primer sequence used for *iNOS* forward was: 5'-AGAGACGCTTCTGAGGTTC-3'; *iNOS* reverse: 5'-TTGATGCTTGTGACTCTTAGG-3'; β -actin (as an internal control) forward: 5'-ATCAGCAAGCAGGAGTACGAT-3'; and β -actin reverse: 5'-AAAGGGTGTAAAACGCAGCTC-3'. For evaluation of the relative gene expression the 2 ${}^{-\Delta \Delta ct}$ method was used (15).

### Determination of IL-1 β protein level by ELISA

The IL-1 β level in the testis homogenates was evaluated using the rat IL-1 β ELISA kit (IBL International, Hamburg, Germany) according to the manufacturer's instructions.

### Sperm analysis

After sacrificing the rat, the epididymis was removed. The end of the epididymis was separated and cut into tiny pieces in Ham's F10, pre-warmed to 37°C for 20 min. In this study, the sperm count, progressive motility, viability, and morphology were determined by using the usual procedures. Sperm counts were done using Lam Neobar and the results were explicated as millions/ml. The viability of sperm was measured using Eosin stain. Sperm samples were blended with an equal volume of 4% eosin Y stain on a glass microscope lam and examined using a light microscope at x400 magnification. The dead sperms were stained red. Sperm cells were counted to acquire the live/dead ratio. Progressive sperm motility was measured using Tris-buffer solution immediately after the collection of the sperm. At first, the percentage of sperm motility was estimated in three microscopic fields and then their mean was recorded as the percentage of sperm motility (x400 magnification). To assess the percentage of morphologically abnormal or normal sperm, the state of the sperm's head, neck, and tail and the total abnormality were examined (16).

### Histological study

To prepare samples of histological study, one testis was detached rapidly, washed by phosphate-buffered saline, fixed in 10% formalin followed by dehydration and embedding in ethanol, xylene, and paraffin, sequentially. Afterward, the tissues segments prepared using microtome were stained by hematoxylin and eosin (17).

### Ethical considerations

The animals were housed in standard cages (12-hr light/dark cycles, constant temperature of 25 ± 2°C, and free access to standard rodent chow and water). These animals were acclimatized for at least 2 wk under these conditions before beginning the experiments. This study was approved by the Research Ethics Committee of Hamadan University of Medical Sciences, Hamadan, Iran (Code: IR.UMSHA.REC.1394.584). The study was performed following the guidelines approved by the Research Ethics Committee of Hamadan University of Medical Sciences, Hamadan, Iran.

### Statistical analysis

The statistical analysis was performed using the SPSS software package version 16.0 (SPSS Inc., Chicago, IL, USA). All results were presented as mean ± SD. To compare the data between different groups, one-way ANOVA analysis, followed by post-hoc Tukey's test were used. A p-value < 0.05 was considered significant.

## 3. Results

### Effects of garlic extract on body weight of rats

Table I presents the body weight of the rats. Initial body weights were similar in all groups (p > 0.05), but the final body weights of the rats were significantly different (p < 0.05). Normal rats gained significant weight throughout the experimental period, while the body weights in both diabetic groups (D1 and D2) decreased significantly when compared with the control group (p = 0.01); however, the final body weights of the D1 + G and D2 + G groups were significantly higher than those of the D1 and D2 groups, respectively (p = 0.01, p = 0.04, respectively).

### Effects of garlic extract on serum glucose level

Table II summarizes the glucose levels during the treatment. Glucose levels were not significantly different in the rats that received aqueous garlic extract in comparison with the control rats (p > 0.05). In diabetic rats (D1 and D2), glucose levels were significantly higher on the 10 ${}^{\mathrm{th}}$ , 20 ${}^{\mathrm{th}}$ , 30 ${}^{\mathrm{th}}$ , and 40 ${}^{\mathrm{th}}$ days after the induction of diabetes when compared with the control group (p = 0.001). The level of glucose was significantly different at the aforementioned four times in the D1 vs. the D2 groups (p = 0.03). Only the oral administration of garlic extract on the 40 ${}^{\mathrm{th}}$ day of treatment was associated with a meaningfully lower glucose level in the D1 + G and D2 + G groups compared with the D1 and D2 rats, respectively (p = 0.018, p = 0.03, respectively).

### Effects of garlic extract on serum testosterone level

**Figure 1:**
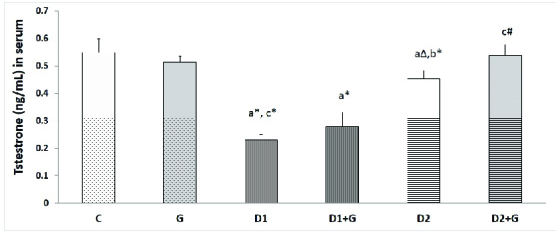
Effects of aqueous garlic extract on the level of testosterone in serum of rats. Results presented as Mean ± SD (n = 6) using one-way ANOVA followed by Tukey's test (p < 0.05). C: Control rats, G: Rats that received aqueous garlic extract, D1: STZ-induced diabetic rats, D1 + G: STZ-induced diabetic rats treated with aqueous garlic extract, D2: STZ + nicotinamide-induced diabetic rats, D2 + G: STZ + nicotinamide-induced diabetic rats treated with aqueous garlic extract, ${}^{a}$ Compared with control rats, ${}^{b}$ Compared with D1, ${}^{c}$ Compared with D2. ${}^{*}$ P < 0.01, ${}^{\#}$ P = 0.01, ${}^{\Delta }$ P = 0.04.

The testosterone level was significantly lower in the D1 and D2 groups compared to the control group (p = 0.001 and p = 0.04, respectively). Also, the D1 group had marginally lower testosterone levels compared with the D2 group (p = 0.001). The level of testosterone in the D1 + G group was higher than that of the D1 rats but this difference was not significant (p > 0.05); however, these levels were significantly different from those of the control rats (p = 0.001). In the D2 + G group, the testosterone level was significantly higher than in the D2 group (p = 0.01) and was near to normal levels in the control rats (Figure 1).

### Effects of aqueous garlic extract on oxidative stress parameters in testis tissues

Table III presents the results of the oxidative stress parameters in the testis tissues. The TAC levels were notably lower in the D1 and D2 groups compared to the control group (p = 0.001). Treatment with oral administration of garlic was associated with a significant enhancement in TAC levels in the D1 + G and D2 + G groups compared with the D1 and D2 groups, respectively (p = 0.006 and p = 0.001, respectively). The TOS level was markedly higher in the D1 and D2 groups compared with the control rats (p = 0.001). TOS levels in the D2 group were significantly higher (p = 0.001) in comparison with the D1 group. Treatment with oral administration of garlic was associated with notably lower TOS levels in the D1 + G and D2 + G groups compared with the D1 and D2 rats, respectively (p = 0.001). The LPO level was significantly higher in the D1 and D2 groups compared to in the control rats (p = 0.001). The LPO level in the D2 group was significantly higher (p = 0.001) in comparison with the D1 group. Treatment with oral administration of garlic was associated with markedly lower LPO levels in the D1 + G and D2 + G groups compared with the D1 and D2 rats, respectively (p = 0.006 and p = 0.001, respectively). There was a notably lower thiol group level in the D1 and D2 groups compared with the control group (p = 0.001). Treatment with oral administration of garlic was associated with a significantly higher thiol group level in the D1 + G and D2 + G rats compared to the D1 and D2 groups, respectively (p = 0.014, p = 0.001, respectively).

### Effects of aqueous garlic extract on *iNOS* gene expression in testis tissues

**Figure 2:**
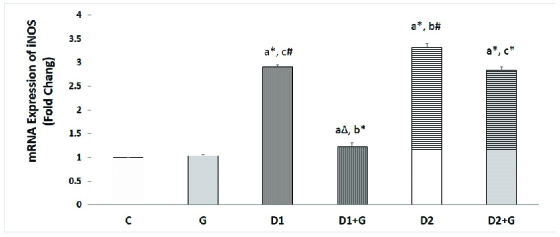
Effects of aqueous garlic extract on *iNOS* gene expression in testis tissues of rats. Results presented as Mean ± SD (n = 6) using one-way ANOVA followed by Tukey's test (p < 0.05). C: Control rats, G: Rats that received aqueous garlic extract, D1: STZ-induced diabetic rats, D1 + G: STZ-induced diabetic rats treated with aqueous garlic extract, D2: STZ + nicotinamide-induced diabetic rats, D2 + G: STZ + nicotinamide-induced diabetic rats treated with aqueous garlic extract. *iNOS*: Inducible nitric oxide synthase. ${}^{a}$ Compared with control rats, ${}^{b}$ Compared with D1, ${}^{c}$ Compared with D2. ${}^{*}$ P < 0.01, ${}^{\#}$ P = 0.01, ${}^{\Delta }$ P = 0.04.

In the D1 and D2 groups, the *iNOS* mRNA level was meaningfully higher in comparison with the control group (p = 0.001) (+2.9 and +3.3, respectively). The level of *iNOS* mRNA in the D2 group was significantly higher than in the D1 group (p = 0.01). Treatment with oral administration of garlic was associated with a notably lower *iNOS* mRNA level in the D1 + G and D2 + G rats in comparison with the D1 and D2 groups (p = 0.001) (-2.36 and -1.16, respectively); however, this difference was more substantial in D1 + G rats than in the D2 + G group (Figure 2).

### Effects of aqueous garlic extract on the protein levels of IL-1 β in testis tissues 

**Figure 3:**
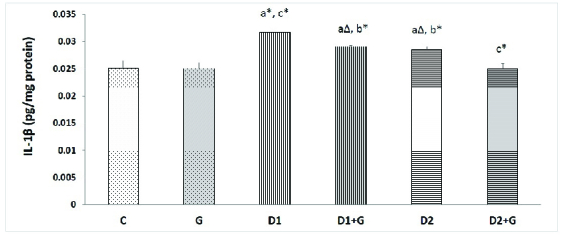
Effects of aqueous garlic extract on the protein levels of interleukin-1 beta (IL-1 β ) in testis tissues of rats. Results presented as Mean ± SD (n = 6) using one-way ANOVA followed by Tukey's test (p < 0.05). C: Control rats, G: Rats that received aqueous garlic extract, D1: STZ-induced diabetic rats, D1 + G: STZ-induced diabetic rats treated with aqueous garlic extract, D2: STZ + nicotinamide-induced diabetic rats, D2 + G: STZ + nicotinamide-induced diabetic rats treated with aqueous garlic extract. ${}^{a}$ Compared with control rats, ${}^{b}$ Compared with D1, ${}^{c}$ Compared with D2. ${}^{*}$ P < 0.01, ${}^{\Delta }$ P = 0.04.

The protein level of Il-1 β in the D1 and D2 groups was significantly higher compared with the control group (p = 0.001 and p = 0.04, respectively). This protein level in the D1 group was significantly higher than in the D2 group (p = 0.001). Oral administration of garlic was associated with notably lower Il-1 β protein levels in D1 + G and D2 + G rats in comparison with the D1 and D2 groups, respectively (p = 0.001); however, this difference was more substantial in the D2 + G rats because the level of this protein was at normal levels in the control rats (Figure 3).

### Effects of aqueous garlic extract on sperm parameters

Table IV summarizes the results of the sperm parameters. Sperm count was significantly lower in the D1 and D2 groups in comparison with the control rats (p = 0.001). The sperm count was higher in the D1 + G group compared with the D1 group (p = 0.02); however, the values of these parameters in the D1 + G and D2 + G groups were still significantly lower than in the control group (p = 0.001). Normal morphology and viability of sperm were significantly lower in D1 rats than in the control and D2 groups (p = 0.001). However, there was no difference between the D2 and control groups (p > 0.05). Treatment with oral administration of garlic was associated with a significantly better normal morphology and viability in the D1 + G group compared to the control group (p = 0.001). The progressive movement in D1 rats was significantly lower compared to the control group (p = 0.001). Also, there was marginally lower progressive movement in the D1 group compared with the D2 rats (p = 0.003). Treatment with garlic was associated with notably better progressive movement in the D1 + G group compared with the D1 group (p = 0.04). However, this parameter in the D1 + G and D2 + G groups was still significantly lower than in the control group (p = 0.003 and p = 0.04, respectively). Motility was markedly lower in the D1 and D2 groups compared with the control group (p = 0.001 and p = 0.018, respectively). Also, there was significantly lower motility in the D1 group compared with D2 rats (p = 0.003). Consumption of garlic extract in diabetic rats was not significantly associated with this parameter; however, the motility of sperm in the D1 + G and D2 + G rats was still significantly different from the control group (p = 0.001 and p = 0.003, respectively).

### Effects of aqueous garlic extract on the histology of testis tissues

**Figure 4:**
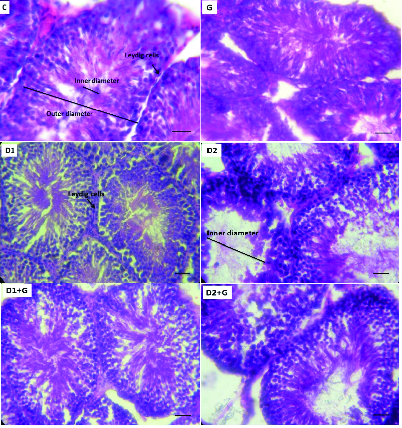
Effects of aqueous garlic extract on the histology of testis tissues of rats stained with hematoxylin and eosin ( × 400).

Figure 4 illustrates the histology of the testis tissues of the rats. In the C and G groups, the structure and size of the inner and outer diameters of the seminiferous tubules were normal. Also, spermatogonia and spermatocytes were observed in normal positions and no fibrosis was observed. In the D1 group, the number of Leydig cells was higher in comparison with the control group. Also, the number of spermatogonia and spermatocytes were lower, although the internal diameter was not different from the control group. In the D2 group, the number of spermatogonia and spermatocytes were lower compared to the control group. Also, the seminiferous tubules were dilated and the inner diameter was longer. In the garlic-treated diabetic rats (D1 + G and D2 + G), the structure of the seminiferous tubules was marginally better compared with the D1 and D2 groups. Also, the number of Leydig cells was lower and the inner lumen was less dilated.

**Table 1:** Effects of aqueous garlic extract on body weight


**Groups**	**Initial body weight (gr)**	**Final body weight (gr)**	** Δ body weight (gr)**
**C**	266 ± 10	309 ± 11	42 ± 6
**G**	285 ± 12	301 ± 13	20 ± 11
**D1**	272 ± 21	251 ± 19 ${}^{a\#}$	-15 ± 8 ${}^{a\#}$
**D1 + G**	251 ± 20	270 ± 14 ${}^{b\#}$	18 ± 14 ${}^{b\#}$
**D2**	271 ± 17	265 ± 21 ${}^{a\#}$	-1 ± 8 ${}^{a\#}$
**D2 + G**	266 ± 8	301 ± 25 ${}^{c\Delta }$	31 ± 21 ${}^{c\Delta }$
Data presented as Mean ± SD. One-way ANOVA followed by Tukey's test, C: Control rats, G: Rats that received aqueous garlic extract, D1: STZ-induced diabetic rats, D1 + G: STZ-induced diabetic rats treated with aqueous garlic extract, D2: STZ + nicotinamide-induced diabetic rats, D2 + G: STZ + nicotinamide-induced diabetic rats treated with aqueous garlic extract. ** Δ ** Body weight: Final body weight-initial body weight. ${}^{a}$ Compared with control rats, ${}^{b}$ Compared with D1, ${}^{c}$ Compared with D2. ${}^{\#}$ P = 0.01, ${}^{\Delta }$ P = 0.04			

**Table 2:** Effects of aqueous garlic extract on serum glucose (mg/dL)


**Groups**	**10 ${}^{\mathrm{th}}$ day**	**20 ${}^{\mathrm{th}}$ day**	**30 ${}^{\mathrm{th}}$ day**	**40 ${}^{\mathrm{th}}$ day**
**C**	85 ± 8	86 ± 6	86 ± 6	89 ± 10
**G**	83 ± 3	84 ± 3	86 ± 3	90 ± 10
**D1**	558 ± 32 ${}^{a*,c*}$	541 ± 76 ${}^{a*,c€}$	521 ± 101 ${}^{a*,c€}$	485 ± 113 ${}^{a*,c€}$
**D1 + G**	545 ± 57 ${}^{a*}$	579 ± 37 ${}^{a*}$	495 ± 73 ${}^{a*}$	321 ± 27 ${}^{a*,b\Delta }$
**D2**	367 ± 102 ${}^{a*,b*}$	398 ± 156 ${}^{a*,b€}$	377 ± 183 ${}^{a*,b€}$	327 ± 140 ${}^{a*,b€}$
**D2 + G**	345 ± 19 ${}^{a*}$	302 ± 142 ${}^{a*}$	262 ± 134 ${}^{a\Delta }$	200 ± 74 ${}^{c€}$
Results presented as Mean ± SD. One-way ANOVA followed by Tukey's test (p < 0.05), C: Control rats, G: Rats that received aqueous garlic extract, D1: STZ-induced diabetic rats, D1 + G: STZ-induced diabetic rats treated with aqueous garlic extract, D2: STZ + nicotinamide-induced diabetic rats, D2 + G: STZ + nicotinamide-induced diabetic rats treated with aqueous garlic extract, ${}^{a}$ Compared with control rats, ${}^{b}$ Compared with D1, ${}^{c}$ Compared with D2. ${}^{*}$ P < 0.01, ${}^{\Delta }$ P = 0.02, ${}^{€}$ P = 0.03				

**Table 3:** Effects of aqueous garlic extract on oxidative stress parameters in testis tissues


**Groups**	**TAC (μmol/mg protein)**	**TOS** **(μmol/mg protein)**	**LPO** **(μmol/mg protein)**	**This group**
**C**	0.0061 ± 0.0002	0.0029 ± 0.0006	0.171 ± 0.198	0.060 ± 0.009
**G**	0.0047 ± 0.0016	0.0024 ± 0.0002	0.220 ± 0.023	0.061 ± 0.013
**D1**	0.0020 ± 0.0009 ${}^{a*}$	0.0151 ± 0.0015 ${}^{a*,c*}$	1.275 ± 0.217 ${}^{a*,c*}$	0.038 ± 0.007 ${}^{a*}$
**D1 + G**	0.0045 ± 0.0011 ${}^{a*,b*}$	0.0016 ± 0.0003 ${}^{b*}$	0.838 ± 0.044 ${}^{a*,b*}$	0.052 ± 0.007 ${}^{b\Delta }$
**D2**	0.0025 ± 0.0009 ${}^{a*}$	0.2520 ± 0.0040 ${}^{a*,b*}$	3.051 ± 0.428 ${}^{a*,b*}$	0.032 ± 0.004 ${}^{a*}$
**D2 + G**	0.0057 ± 0.0016 ${}^{c*}$	0.0020 ± 0.0005 ${}^{c*}$	0.250 ± 0.036 ${}^{c*}$	0.052 ± 0.005 ${}^{c*}$
Results presented as Mean ± SD (n = 6) using one-way ANOVA followed by Tukey's test (p < 0.05), C: Control rats, G: Rats that received aqueous garlic extract, D1: STZ-induced diabetic rats, D1 + G: STZ-induced diabetic rats treated with aqueous garlic extract, D2: STZ + nicotinamide-induced diabetic rats, D2 + G: STZ + nicotinamide-induced diabetic rats treated with aqueous garlic extract. TAC: Total antioxidant capacity, TOS: Total oxidative status, LPO: Lipid peroxidation. ${}^{a}$ Compared with control rats, ${}^{b}$ Compared with D1, ${}^{c}$ Compared with D2. ${}^{*}$ P < 0.01, ${}^{\Delta }$ P = 0.01				

**Table 4:** Effects of aqueous garlic extract on sperm parameters


**Groups**	**Sperm count (millions/mL)**	**Normal morphology %**	**Viability %**	**Progressive movement %**	**Motility %**
**Control**	277 ± 24.85	96.2 ± 1.81	97.4 ± 1.26	57 ± 7.3	73.8 ± 6.19
**G**	260 ± 35.77	96.33 ± 1.36	97 ± 0.89	48 ± 4.73	60.66 ± 6.28
**D1**	86.66 ± 34.33 ${}^{a*}$	89.16 ± 4.23 ${}^{a*,c*}$	92.83 ± 2.2 ${}^{a*,c*}$	18 ± 7.57 ${}^{a*,c}$	29.5 ± 8.52 ${}^{a*,c*}$
**D1+G**	148 ± 91.74 ${}^{a*,b\Delta }$	95.60 ± 2.54 ${}^{b*}$	97.6 ± 1.07 ${}^{b*}$	32.4 ± 17.93 ${}^{a*,b€}$	38.8 ± 16.52 ${}^{a*}$
**D2**	93.33 ± 27.9 ${}^{a*}$	97 ± 2.17 ${}^{b*}$	96.66 ± 1.55 ${}^{b*}$	39.16 ± 16.73 ** ${}^{b\neq }$ **	48.5 ± 17.79 ${}^{a\Delta ,b*}$
**D2+G**	105.83 ± 15.78 ${}^{a*}$	96.33 ± 1.36	97.33 ± 1.55	39.83 ± 10.38 ** ${}^{a€}$ **	48.66 ± 9.56 ${}^{a*}$
Results presented as mean ± SD (n = 6) using one-way ANOVA followed by Tukey's test (p < 0.05), C: Control rats, G: Rats that received aqueous garlic extract, D1: STZ-induced diabetic rats, D1 + G: STZ-induced diabetic rats treated with aqueous garlic extract, D2: STZ + nicotinamide-induced diabetic rats, D2 + G: STZ + nicotinamide-induced diabetic rats treated with aqueous garlic extract. ${}^{a}$ Compared with control rats, ${}^{b}$ Compared with D1, ${}^{c}$ Compared with D2. ${}^{*}$ P < 0.01, ${}^{\Delta }$ P = 0.02, ${}^{€}$ P = 0.04					

## 4. Discussion

DM is one of the most common metabolic diseases and its prevention is crucial due to its high prevalence and complications. Diabetes can affect several organs. Clinical and laboratory studies have shown that diabetes affects the male reproductive system. This metabolic disease impairs sexual function by decreasing testosterone levels, the diameter of tubes, libido, sperm motility, and sperm count. For this reason, diabetes is identified as a cause of infertility (1, 3). In this study, testicular complications of diabetes were investigated in an animal model. We used STZ for induction of type-1 diabetes (D1) and STZ + nicotinamide for the induction of a mild form of this disease, similar to type-2 diabetes (D2) in rats.

In diabetes, persistent blood glucose leads to the production of free radicals such as ROS and NO. Oxidative stress is a strong intermediary in cell death (18). Hyperglycemia also activates macrophages and elevates inflammatory cytokine levels in testis tissues (19, 20). The results of this study showed that the final body weight and levels of testosterone, TAC and thiol group, and the quality of sperm were lower in diabetic rats (D1 and D2) compared with the control group, whereas the levels of glucose, TOS, LPO, *iNOS* gene expression, and IL-1 β protein were higher in diabetic rats (D1 and D2) compared with the control rats. These results confirm that diabetes can induce oxidative stress and inflammation in the testis tissues of diabetic rats.

Drug therapy is effective for long-term metabolic control of DM for the reduction of its complications. Today, most drugs that regulate blood glucose and reduce the complications of this disease are insulin therapy and hypoglycemic drugs. Because hyperglycemia-induced oxidative stress and inflammation are the main causes of diabetic complications, each compound that can reduce blood glucose or has antioxidant and anti-inflammatory properties can inhibit this pathway. In recent years, researchers have used herbal medicine for the treatment of various diseases because they are inexpensive and have few side effects, although the action mechanism of such medicines is often unclear (21). The effects of different plant extracts on testis tissues in diabetic rats have been investigated (22, 23). Garlic is one of the herbs used in traditional medicine, such as diabetes therapy. Research has shown that the allicin in garlic can trap free radicals, inhibit LPO, and reduce blood lipids. Other studies have shown that the use of garlic can increase the amount of testosterone, due to a reduction in oxidative stress (24).

The results of this study indicated a lower level of blood glucose in garlic-treated diabetic rats (D1 + G and D2 + G) in comparison with untreated diabetic rats. The hypoglycemic activity of garlic mainly relates to its allicin components. Allicin diminishes blood glucose by increasing the secretion of endogenous insulin or increasing the sensitivity of target tissue to insulin (25). Several studies have shown the hypoglycemic effects of garlic extract (26, 27).

The results of the current study demonstrated a considerable gain in the body weight of garlic-treated diabetic rats in comparison with the untreated diabetic groups. Following the rats' treatment with garlic, their weight increased to a similar level as the control group. This increase may be due to garlic's insulin-like function and its promotion of increased glucose transporter-4 activity (28, 29).

DM has several effects on reproductive and sexual activity in humans and animals. Insulin deficiency in diabetes is related to disorders of Sertoli and Leydig cell activities, which, as a result, affects sexual function (30). DM can reduce the number of Leydig cells and their activity. Additionally, a decrease in the expression of GLUT-3 and androgen receptors and insulin - like growth factor-I can cause their dysfunction. These changes reduce serum testosterone levels and, as a result, decrease the number of spermatogenic cells (31). The results of this study showed lower testosterone levels in both diabetic groups compared to the control rats. Since diabetes-induced oxidative stress leads to a reduction in testosterone levels (32), treatment with antioxidant agents can improve these levels. In this study, we used garlic as an antioxidant for the treatment of diabetic rats. Testosterone levels in both of the garlic-treated diabetic rat groups (D1 + G and D2 + G) were higher in comparison with the untreated diabetic groups.

The antioxidant properties of garlic and their effect on different organs have been investigated in many studies (33-36). Testicular tissue is highly sensitive to free radicals and oxidative stress because the tissue has a high cell division rate. It is postulated that increases in oxidative stress can lead to reduced testosterone secretion by testicular Leydig cells (37). Furthermore, the usage of antioxidant supplements is recommended for neutralizing free radicals, decreasing oxidative stress, and enhancing spermatogenesis and fertility (20). Garlic, due to its antioxidant properties, is a good candidate for this purpose. Our results showed higher levels of TAC and the thiol group and lower levels of TOS and LPO in the testis tissues of garlic-treated diabetic rats in comparison with the untreated diabetic groups. Thus, these results demonstrate the antioxidant activity of garlic.

In diabetic patients, oxidative stress increases significantly and NO levels in the plasma are higher than in healthy patients (34). *iNOS* is an important enzyme in the NO synthesis pathway. Sönmez et al. showed that the gene expression of *iNOS* increased in the testis tissues of STZ-diabetic rats (38). The results of our study showed that the expression of this gene was significantly higher in both diabetic groups (D1 and D2) compared with the control rats. Treatment of diabetic rats with garlic extract was associated with remarkably lower *iNOS* gene expression in comparison with the untreated diabetic rats. Some studies have shown that glutathione plays a role in the regulation of *iNOS* expression so that a reduction in glutathione leads to an increase in *iNOS* gene expression level (39). Therefore, any factor that increases the level of reduced glutathione can enhance the expression of the *iNOS* gene. Since garlic increases the level of reduced glutathione (40), the higher level of *iNOS* expression observed in the groups that received garlic extract was expected.

Chronic hyperglycemia can induce oxidative stress and activate NF-κB. By activating this factor, the expression of several genes involved in inflammation are upregulated. Some researchers have reported high levels of inflammatory factors such as tumor necrosis factor-alpha (TNF-α), IL-6, and IL-1 β in diabetic subjects and animal models (41). In this study, our results showed a higher level of IL-1 β protein in the testis tissues of both diabetic groups (D1 and D2) in comparison with the control rats, which confirmed the presence of inflammation in these tissues, whereas the level of this protein was lower in diabetic rats treated by oral administration of garlic extract compared with the untreated diabetic rats. So, it seems that garlic, due to its antioxidant properties, can reduce the activity of NF-κB, thereby reducing inflammatory cytokines such as IL-1 β .

Additionally, garlic extract treatment was associated with better semen parameters in garlic-treated diabetic rats compared with D1 and D2 rats. The results of the histopathology analyses supported the biochemical and molecular results.

## 5. Conclusion

In garlic-treated diabetic rats, garlic extract was associated with reduced glucose levels, oxidative stress, IL-1 β levels, and gene expression of *iNOS* and increased testosterone levels and sperm quality. So, the results suggest that garlic can help to reduce the severity of damage in the testicular tissues of diabetic rats through its hypoglycemic, antioxidant, and anti-inflammatory properties.

##  Conflict of Interest 

No potential conflict of interest relevant to this article was reported.
